# Composition of the white precipitate formed on the surface of damaged triacetyl cellulose-based motion picture films

**DOI:** 10.1038/s41598-020-80498-5

**Published:** 2021-01-15

**Authors:** Keiko Takahashi, Maiko Sasaki, Hiroshi Hayakawa, Hitoshi Yajima, Yoshiki Oda

**Affiliations:** 1 Faculty of Engineering, Tokyo Polytechnic University, 1583 Iiyama, Atsugi, Kanagawa 243-0297 Japan; 2 Technology Joint Management Office, Tokai University, 4-1-1 Kitanakame, Hiratsuka, Kanagawa 259-1292 Japan

**Keywords:** Analytical chemistry, Materials chemistry, Chemistry, Materials science

## Abstract

To achieve a better understanding of the “vinegar syndrome” phenomenon, which has caused serious damage to triacetyl cellulose-based motion picture films, the white powder obtained from damaged film surfaces was analysed in this study. The powder was found to be soluble in acetone, diethyl ether, dimethylformamide, and chloroform, but insoluble in water. From the results of ^1^H, ^13^C and ^31^P nuclear magnetic resonance spectroscopy, mass spectrometry, and X-ray fluorescence measurements, it was concluded that the white precipitate had a molecular weight of 326 amu and was composed of triphenyl phosphate (C_18_H_15_O_4_P).

## Introduction

Nowadays, images are digitised, and photos and motion pictures are rarely recorded on films. However, many old photographic and motion picture films have been preserved as the cultural heritage. From the viewpoint of stable preservation of valuable cultural artefacts, possible film deterioration must be timely detected. Most of the currently stored films are fabricated from cellulose acetate (CA)^1,2^, which is a versatile semi-synthetic plastic material widely utilised in the twentieth century. Triacetyl cellulose (TAC), which contains no free hydroxyl groups (its degree of acetyl substitution is equal to 3.0) represents a typical CA derivative. It has been used as the base material for photographic, motion picture, and microscopic films since the development of so-called safety films in the 1920s as a replacement for the unstable and highly flammable cellulose nitrate base. TAC is still employed in optical films such as protective coatings for the polarising panels of liquid crystal television displays, notebook computers, and mobile phones. CA is also widely applied in various fields, including textile fibres, the fleece of cigarettes filters, coating materials, adhesive binders, and inks^3,4^. Nevertheless, the use of TAC as the film base had its disadvantages, such as the hydrolysis caused by the film storage under hot and humid conditions, which was the first instance of TAC degradation reported by the Indian government within a decade after its introduction in 1948^5^. Afterwards, other cases of film degradation were reported for collections stored under similar conditions. Since the 1980s, serious film stability concerns have been expressed as a result of such reports. It was observed that film exposures to moisture, heat, and acids could lead to the deacetylation of their TAC bases^6–10^. The related degradation reaction was called a *vinegar syndrome*, which was accompanied by vinegar odour, film shrinkage, embrittlement, buckling of the gelatine emulsion, and the formation of a white precipitate on the film surface. During the advanced stages of the deterioration process, the degree of film shrinkage could reach 10%. However, its gelatine emulsion did not shrink with the film as it did not undergo deterioration, leading to the separation of the emulsion from the film base (a so-called “channelling” effect). Once this process has begun, the remaining life of the film was shortened significantly due to the acceleration and autocatalysis of the degradation reaction^11–14^.

To prevent such degradation, early diagnosis of the films stored under cool and humid conditions is required^10,15–22^. Although standard storage conditions for TAC-based films have been established, chemical analysis of the degradation products was not performed, and efficient diagnostic techniques were not developed. Recently, the vinegar syndrome has been investigated in more detail due to the significant advances in the field of analytical instrumentation^22–24,26–30^. In particular, the degradation of CA museum artefacts was examined by micro-Fourier transform infrared (FTIR) spectrometry, ion chromatography, and X-ray fluorescence (XRF) spectrometry^23^. In addition, special anti-hydrolysis agents were identified in a study conducted using thermal analysis and high-performance liquid chromatography techniques^24^. It was found that the degradation process caused film shrinkage and increased film brittleness, owing to the hydrolysis of acetyl ester, glycoside bond cleavage, and loss of plasticisers.

As TAC is brittle and thermally decomposes prior to reaching its softening point, plasticisers must be added to reduce the glass transition temperature of the polymer matrix and improve its physical properties, such as stability, workability, flexibility, and tensile strength^4^. Typical plasticisers compatible with CA include citrates, phthalates (such as dibutyl phthalate, diethyl phthalate, dimethyl phthalate, di-2-methoxyethyl phthalate, ethyl phthalyl ethyl glycolate, and methyl phthalyl ethyl glycolate), and phosphates (such as triethyl phosphate and triphenyl phosphate (TPP)). For example, 10–20 wt% triphenyl phosphate and 20–40 wt% phthalate esters are often utilised for the qualitative and quantitative production of films and sheets^25^. In addition, plasticiser losses were estimated by gas chromatography^26,29,30^, mass spectrometry (MS)^22,29^, FTIR spectroscopy^22,29^, and thermal analysis^27,28^. In some cases, the historical TAC-based film material contained several plasticisers^30^.

As mentioned above, the vinegar syndrome remains a serious problem. However, its comprehensive chemical analysis has not been conducted yet. The base layer of the film contains large amounts of plasticisers besides CA. One of the symptoms of the vinegar syndrome is the precipitation of a white solid on the film surface, which is sometimes called “acetic acid powder” by field workers. However, this powder is different from acetic acid, and the correlation between the degree of film deterioration and precipitation of the white solid has not been fully determined.

Therefore, for the purpose of this study, we obtained a number of historical motion picture films with established origins. A detailed investigation of the library was performed, and a white precipitate was collected from the surface of the damaged films. Using the results of our previous works and general analytical methods, we attempted to identify the white precipitate by conducting ^1^H, ^13^C, and ^31^P nuclear magnetic resonance (NMR) spectroscopy, MS, and XRF analyses. This study represents the first step of applying NMR spectroscopy to the chemical examination of the vinegar syndrome.

## Results

### Solubility studies

The results of solubility studies revealed that a 5 mg sample of the white precipitate was soluble in 0.02 mL of *N*,*N-*dimethyformamide, dimethylsulfoxide (DMSO), chloroform, methanol, ethanol, and acetone, but was insoluble in water.

### ^1^H and ^13^C NMR spectroscopy studies

**Figure 1:**
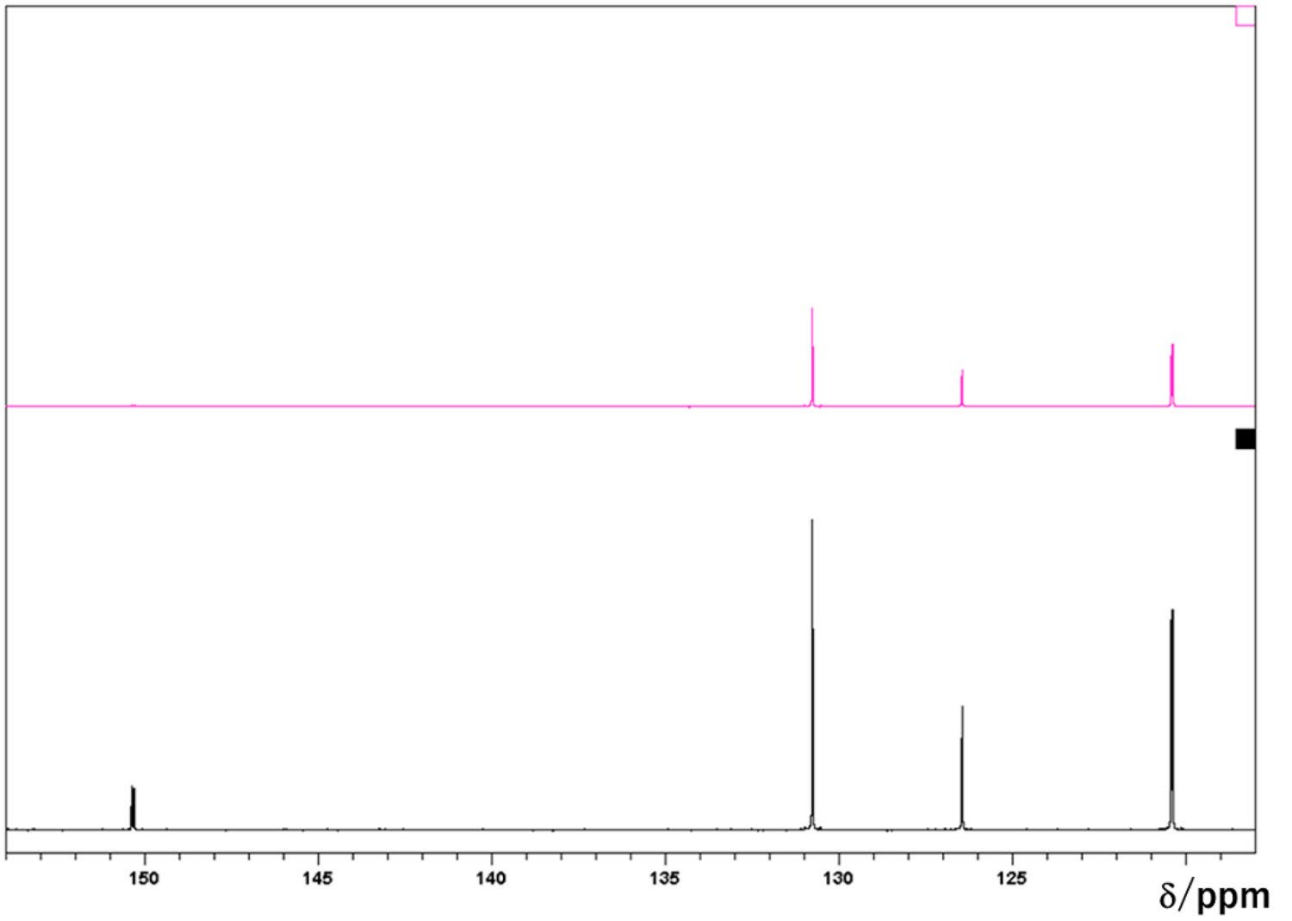
^13^C NMR spectra of the white precipitate: standard ^13^C spectrum (lower panel) and DEPT spectrum (upper panel).

**Figure 2:**
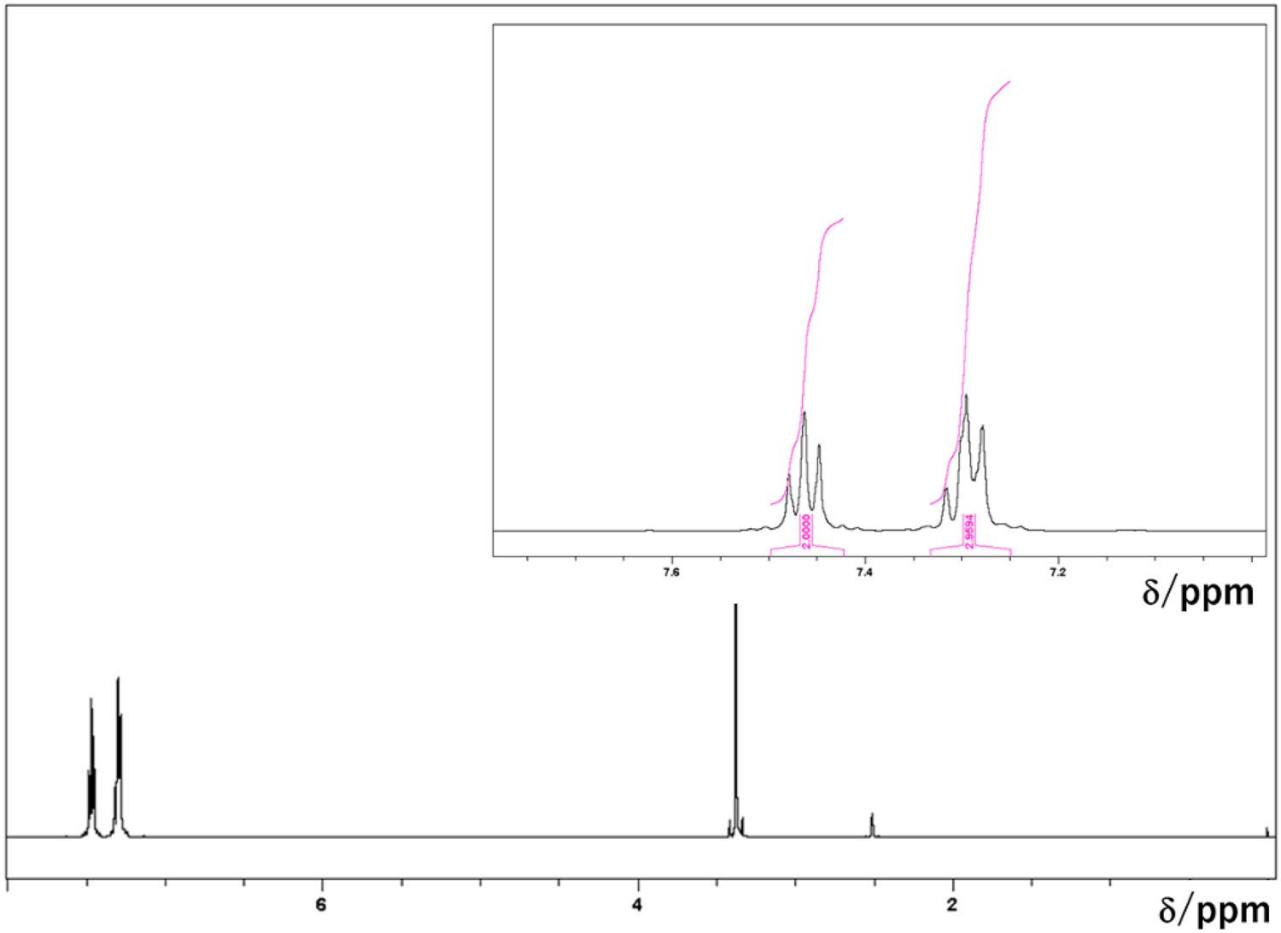
^1^H NMR spectrum of the white precipitate.

**Figure 3:**
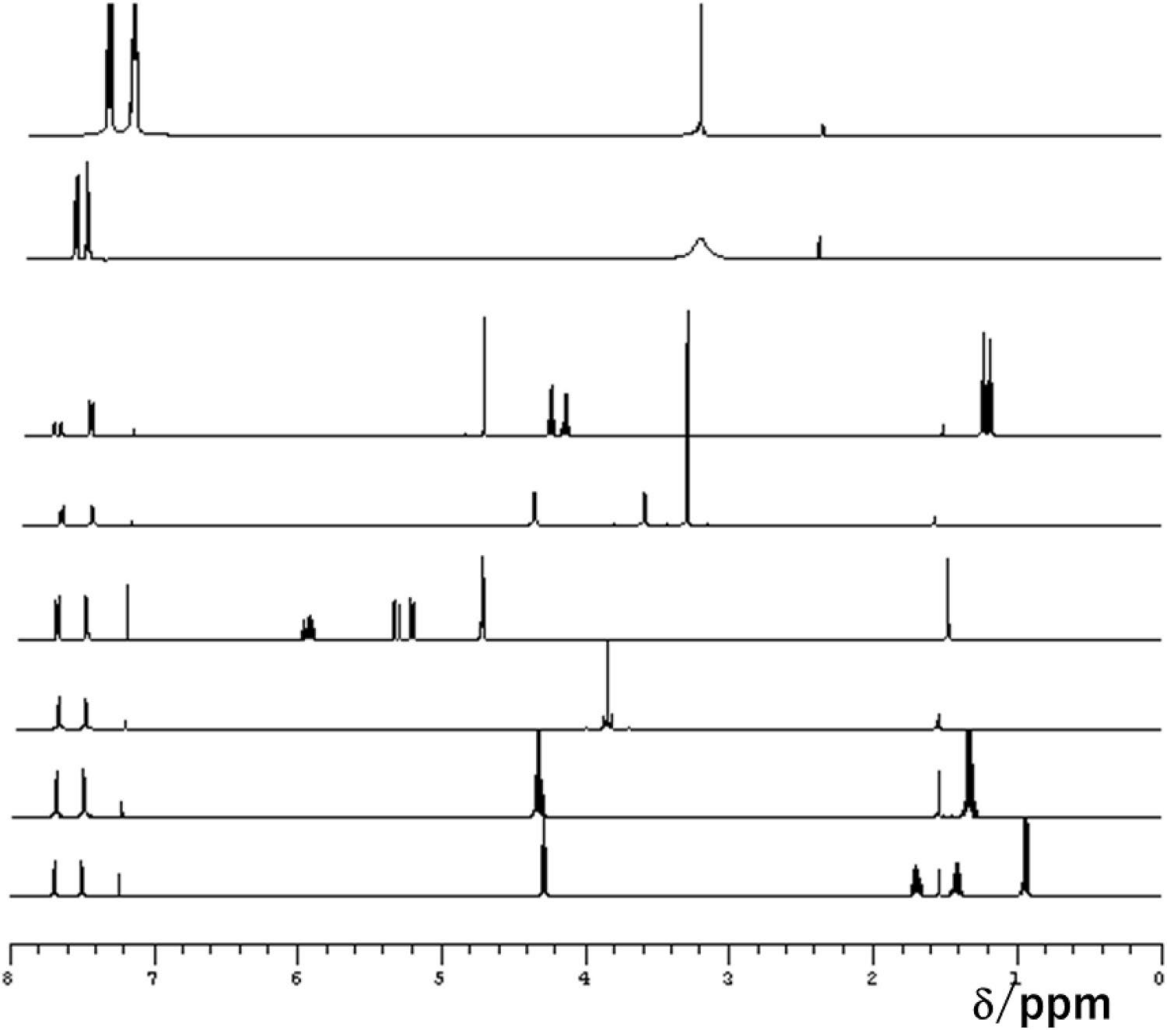
^1^H NMR spectra of (from top to bottom): the white precipitate, phthalic acid, ethyl phthalyl ethyl glycolate, bis(2-methoxyethyl) phthalate, diallyl phthalate, dimethyl phthalate, diethyl phthalate, and dibutyl phthalate in CDCl_3_.

Initially, we analysed the white powder by NMR spectroscopy. In the obtained 1D^13^C NMR spectra, the peaks corresponding to aromatic carbon atoms were observed at 120, 126, and 130 ppm, while the peak representing a single quaternary carbon atom was detected at 150 ppm (Fig. 1). The distortionless enhancement by polarisation transfer (DEPT) spectra also indicated that the white precipitate contained aromatic moieties and a quaternary or carbonyl carbon, but no aliphatic carbon atoms. After the ^13^C NMR analysis, we recorded a ^1^H NMR spectrum of the white powder in DMSO-*d*_6_, which exhibited two aromatic peaks at 7.3 and 7.5 ppm with a 2:3 intensity ratio (Fig. 2). The observed signal pattern in the aromatic region is representative of a mono-substituted benzene ring or phenyl derivative and suggests the presence of a single type of phenyl groups in the precipitate. Except for the peak centred at ~ 3.6 ppm (which was assigned to water), no other features were detected, indicating that the sample did not contain any aliphatic hydrocarbon moieties. In addition, Fig. 3 displays the NMR spectra of phthalic acid (a typical phthalate plasticiser), ethyl phthalyl ethyl glycolate, bis(2-methoxyethyl) phthalate, diallyl phthalate, dimethyl phthalate, diethyl phthalate, and dibutyl phthalate. It shows that the spectrum of the white precipitate powder was comparable to that of phthalic acid, although in the latter case, the aromatic ratio was 2:2 due to the presence of a disubstituted benzene derivative. Moreover, as the plasticisers examined in this work included esters, additional peaks corresponding to aliphatic hydrocarbons were observed as well.

### MS analysis

In the mass spectrum of the white precipitate, the most abundant peak was centred at *m/z* = 349, although a number of weaker peaks were also observed at *m/z* = 327, 350, and 351. For all the tested analytes, higher fragmentation voltages (representing higher electrostatic potentials at the entrance lens of the mass analyser) generally increased the intensities of the characteristic fragment ions proportionally to the decrease in the intensity of the corresponding molecular ion peak. Therefore, it was concluded that the white powder was a single chemical compound and not a mixture of several compounds. As the species analysed in the positive ionization mode are generally observed either in their protonated forms or with sodium, we inferred that the lower mass of 327 corresponded to the protonated molecular ion [M + H]^+^, while the most abundant peak at *m/z* = 349 represented [M + Na]^+^, and those at *m/z* = 350 and 351 resulted from protonated [M + Na]^+^ species. Hence, the molecular weight of the white precipitate was 326 amu.

### XRF and ^31^P NMR measurements

**Figure 4:**
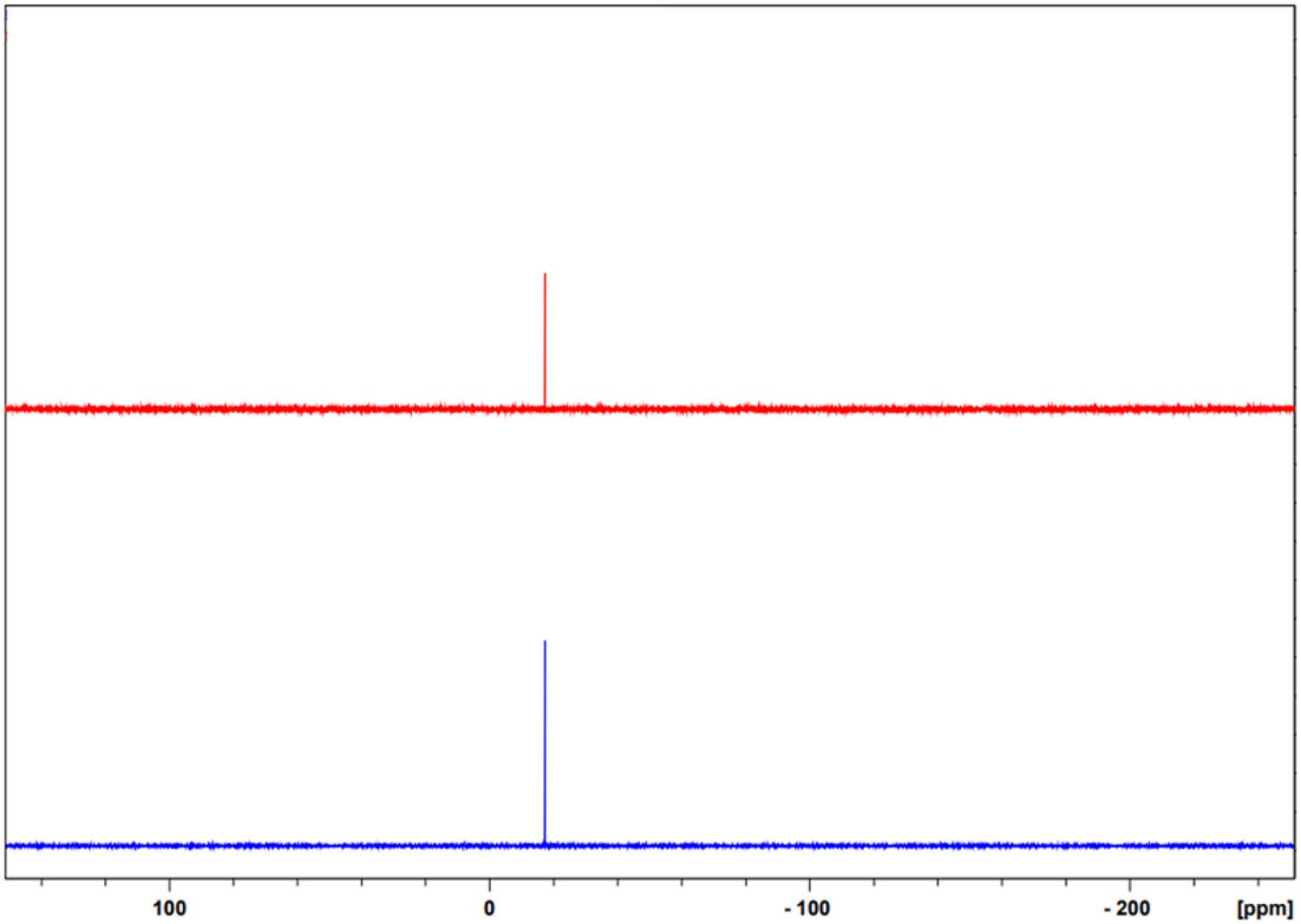
^31^P NMR spectra of the white precipitate (upper) and authentic TPP(lower) in DMSO-*d*_*6*_.

During analysis, all samples were mounted on filter paper, and any detected traces of carbon and oxygen were excluded from the data obtained to identify the white precipitate composition. According to the results of XRF measurements, the certified elements included Al (0.008%), Si (0.03%), P (4%), Cl (0.006%), Fe (0.03%), and Sn (0.1%). Thus, from the NMR and MS spectra discussed above, it was concluded that the white powder was partially composed of phosphorous (2–8 wt% depending on the instrumentation limits) with Al, Si, Cl, Fe, and Sn impurities. In the recorded ^31^P NMR spectrum, a single peak was observed at − 17.26 ppm in DMSO-*d*_*6*_ (Fig. 4), and − 17.68 ppm in CDCl_3._ These single chemical shift values were same authentic TPP’s signal shift, suggesting the presence of a single phosphorus environment.

## Discussion

The white precipitate was found to be soluble in polar organic solvents, and its molecular weight was 326 amu. The ^1^H, ^13^C, and ^31^P NMR spectra indicated that the precipitate contained a single type of phenyl moieties, a quaternary or carbonyl carbon, and phosphorous, but no aliphatic carbon atoms. As the molecular weight of the phenyl group was 78 (or 77 minus 1 hydrogen atom), the white powder could not contain more than four phenyl groups (their actual number was most likely 3 or 4), indicating the presence of an additional fraction that bound phenyl groups together through covalent bonds. The results of NMR spectroscopy, MS, and XRF analyses suggested that the white precipitate was a single compound containing phosphorus in addition to 3–4 phenyl groups, and that its molecular weight was 326. Therefore, we concluded that the analysed white powder was TPP with a calculated phosphorus content of 9.5 wt%. Although the average phosphorus content of 4% (2–8 wt%) was obtained by XRF, the XRF results were within the measurement error range; hence, both the molecular formula and corresponding molecular mass of TPP (C_18_H_15_O_4_P) satisfied the above-mentioned conditions.

The absence of such signals from the spectrum of the white precipitate allowed us to conclude that it did not contain either phthalate plasticisers or phthalic acid^31,32^. Our previous report was incorrect. The obtained ^1^H, ^13^C and 31P NMR spectra also suggested that white powder consisted of pure TPP.

## Concluding remarks

The results of NMR spectroscopy, MS, and XRF studies showed that the white precipitate collected from the motion picture films stored in the library of the Tokyo Polytechnic University was TPP (C_18_H_15_O_4_P). This compound was likely produced by the film itself due to the addition of TPP plasticiser during film preparation. The TAC-based motion picture films also contained other plasticisers (such as phthalates), whose purpose was to enhance their flexibility, flame resistance, and stability with respect to heat, humidity, and pressure. Among the different plasticisers, only TPP diffused from the inside of the film to the surface as the deterioration progressed and then recrystallised. Although we did not know the exact film composition, previous studies indicated that a typical TAC film contained 100 g of CA, 60 g of ethyl or methyl alcohol, and 15 g of TPP^17^. The melting point of TPP was 48–50 °C; therefore, the climate of many countries promoted TPP melting. During the degradation of the motion picture films, deacetylation occurred initially to produce acetic acid. We hypothesise that this process decreases the compatibility of the plasticiser with CA, which may accelerate the TPP removal from the film, resulting in the formation of a brittle film base. Future research studies in this area will focus on the complete chemical analysis of TAC motion picture films, optimisation of their storage conditions, and development of novel strategies for preventing the degradation of aging films and their restoration.

## Methods

### Materials

The films used in this study were previously stored in a research laboratory until 1958, when they were transferred to the Hino factory of Konica Inc., after which they were formally registered in a film library. In 1988, hundreds of film volumes were removed from the library, and the nitrocellulose-based films were incinerated. After the cleaning procedure conducted in March 1990, a selection of 35-mm and 16-mm films were registered and stored in the National Museum of Modern Art, Tokyo National Film Centre. In June 1990, the remaining films were transferred to the Tokyo Polytechnic University in Atsugi. As no vinegar odour was detected at that time, it was assumed that any possible degradation process was in its early stage. The films were then stored in a laboratory without special treatment or control over the humidity and temperature. The film samples (113 volumes) were extracted from the collection for analysis in July 2012. The averaged acetylated number per 1 glucose unit (DS) of the damaged film on which white precipitate existed was 0.87. Over 2 acetylates were hydrolysed^33,34^.

Triphenyl phosphate (TPP) was purchased by Kanto Kagaku Co. Ltd of special grade reagent. The other reagents were also purchased by Kanto Kagaku Co. Ltd of special grade reagents, and used without further purification unless otherwise stated.

### Sampling procedure

A white precipitate was observed on 4 of the 113 film volumes and collected using a microspatula. A persistent vinegar smell was detected, and the surfaces and edges of the films were highly curled. After drying under reduced pressure, the precipitate was analysed as outlined in the following subsections.

### NMR spectroscopy measurements

^1^H, ^13^C, and ^31^P NMR spectra were recorded on a Bruker AVANCE III HD 500 spectrometer at room temperature using DMSO-*d*_*6*_ or CDCl_3_ as the solvent. One-dimensional (1D) ^13^C NMR and DEPT spectra were obtained over a spectral width of 23,148 Hz (a total of 64 k data points were collected). For the 1D ^13^C NMR (125 MHz) and ^31^P NMR (202 MHz) spectra, 1024 and 32 scans were performed, respectively.

### MS measurements

MS spectra were recorded on a JEOL JMS-T100LP electrospray ionisation mass spectrometer in the positive ion mode at room temperature using methanol solvent.

### X-ray fluorescence measurements

This study was conducted at the Toray Research Center, Japan. XRF analysis was performed on a ZSX Primus II spectrometer (Rigaku Co., Japan) equipped with a scintillation detector containing a LiF crystal and a gas flow-type proportional counter containing LiF, Ge, PET, RX25, RX40, and RX61 crystals in vacuo for the detection of 73 elements ranging from _5_B to _92_U. Each element concentration in the sample was estimated between M/2 and 2 M.
